# Flexible Memory Device Composed of Metal-Oxide and Two-Dimensional Material (SnO_2_/WTe_2_) Exhibiting Stable Resistive Switching

**DOI:** 10.3390/ma14247535

**Published:** 2021-12-08

**Authors:** Ghulam Dastgeer, Amir Muhammad Afzal, Jamal Aziz, Sajjad Hussain, Syed Hassan Abbas Jaffery, Deok-kee Kim, Muhammad Imran, Mohammed Ali Assiri

**Affiliations:** 1 Department of Physics & Astronomy and Graphene Research Institute, Sejong University, Seoul 05006, Korea; 2 Department of Physics, Riphah International University, 13-km Raiwind Road, Lahore 54000, Pakistan; Amirafzal461@gmail.com; 3 Department of Electrical Engineering, Sejong University, 209 Neungdong-ro, Gwangjin-gu, Seoul 05006, Korea; azizjamal37@gmail.com (J.A.); deokkeekim@sejong.ac.kr (D.-k.K.); 4 HMC (Hybrid Materials Center), Department of Nanotechnology & Advanced Materials Engineering and Graphene Research Institute, Sejong University, Seoul 05006, Korea; shussainawan@gmail.com (S.H.); hassan@sju.ac.kr (S.H.A.J.); 5 Department of Chemistry, Faculty of Science, King Khalid University, P.O. Box 9004, Abha 61413, Saudi Arabia; imranchemist@gmail.com (M.I.); maassiri@kku.edu.sa (M.A.A.)

**Keywords:** resistive switching, memristor, 2D-materials, flexible devices, transparent

## Abstract

Two-terminal, non-volatile memory devices are the fundamental building blocks of memory-storage devices to store the required information, but their lack of flexibility limits their potential for biological applications. After the discovery of two-dimensional (2D) materials, flexible memory devices are easy to build, because of their flexible nature. Here, we report on our flexible resistive-switching devices, composed of a bilayer tin-oxide/tungsten-ditelluride (SnO_2_/WTe_2_) heterostructure sandwiched between Ag (top) and Au (bottom) metal electrodes over a flexible PET substrate. The Ag/SnO_2_/WTe_2_/Au flexible devices exhibited highly stable resistive switching along with an excellent retention time. Triggering the device from a high-resistance state (HRS) to a low-resistance state (LRS) is attributed to Ag filament formation because of its diffusion. The conductive filament begins its development from the anode to the cathode, contrary to the formal electrochemical metallization theory. The bilayer structure of SnO_2_/WTe_2_ improved the endurance of the devices and reduced the switching voltage by up to 0.2 V compared to the single SnO_2_ stacked devices. These flexible and low-power-consumption features may lead to the construction of a wearable memory device for data-storage purposes.

## 1. Introduction

Two-terminal, non-volatile memory devices are becoming the most effective and notable devices because of their data-storage capability and fast operating speed. Besides these, there are flash memory devices that operate on a charge storage mechanism but have comparatively low operation capabilities [1,2]. Resistive-switching random-access memory (RRAM) provides a substitute for ordinary silicon-based devices, and presents various appealing packages, consisting of: configuration for a simple two-terminal appliance, unmatchable reliability, a less time-consuming operation speed, and reduced power consumption [3,4]. The fundamental working of RRAMs is based on their triggering from a high-resistance state (HRS) to a low-resistance state (LRS), or vice versa, by applying suitable programming voltages. Furthermore, transitional resistance states can likewise be reached, leading to extra functionalities, for example, multibit capacity, mathematical and neuromorphic processing, and data storage [5,6,7]. Until today, a variety of materials have been discovered to display resistive switching, of which the most familiar are transition metal oxides [8], perovskite oxides [3], and chalcogenide phase-change alloys [9].

Resistive-switching properties have also been displayed by different carbon materials, consisting of amorphous carbons [10], oxygenated amorphous carbon [11], and graphene oxide [12]. A few prior studies suggest that graphene oxide (GO), among all the above choices for RRAM, has significantly more precise and viable utilizations on a scientific and commercial scale, due to its significantly lower cost of fabrication, naturally sustainable manufacturing process, and high mechanical flexibility; all of these properties make it a perfect priority for future electronics [12]. However, it is also reported in previous studies that GO memory devices have produced contradictory and inconsistent findings. For instance, a few authors have observed bipolar resistive switching in Au/GO/Pt structures [13], but others have not [14]. Similarly, some structures, such as Al/GO, have displayed perfect memory characteristics on both types of substrate (flexible and rigid) [12]. While for the identical structure, no switching was experienced in further studies [15]. Most commonly, the memory devices switch from the HRS to the LRS (called the SET process) because of metal filament formation [5,14,15,16,17]. These filaments are formed due to the diffusion of the metal ions and their stacking. Materials with a large bandgap and electric resistivity are considered more promising for memory devices [17]. Because of their direct use for biological purposes, flexible gadgets are also in high demand from the wearable device industry. The inadequate adhesive capability of the channel materials, the metal electrodes, and their cracking issues are the main barriers to fabricating the next generation of flexible memory devices. The manufacture of flexible memory devices was the most difficult task for researchers before the discovery of flexible two-dimensional (2D) materials. These two-dimensional materials now have a wide range of applications, including photodetectors, diodes, high-frequency devices, and solar cells [18,19,20,21,22]. 

Here, we introduce a highly stable and flexible memory device composed of a bilayer structure of tin-oxide/tungsten-ditelluride (SnO_2_/WTe_2_) sandwiched between Ag (top) and Au (bottom) metal electrodes over a flexible polyethylene terephthalate (PET) substrate. The flexible nature of the two-dimensional WTe_2_ provides a crack-free, superior platform for the SnO_2_ to fabricate a flexible memory device over the PET substrate. The filament formation, which is responsible for the resistive switching, is explained with its underlying mechanism. Repeatable cycles of resistive switching are reported while changing the thickness of the SnO_2_. A few layers of thick WTe_2_ films provide highly repeatable resistive switching with a thicker SnO_2_. The Ag/SnO_2_/WTe_2_/Au devices exhibited excellent retention and bending endurance. This research might lead to the development of highly stable and flexible resistive-switching memristor devices for next-generation wearable electronics.

## 2. Experimental Details

### Device Fabrication and Characterizations

**Figure 1:**
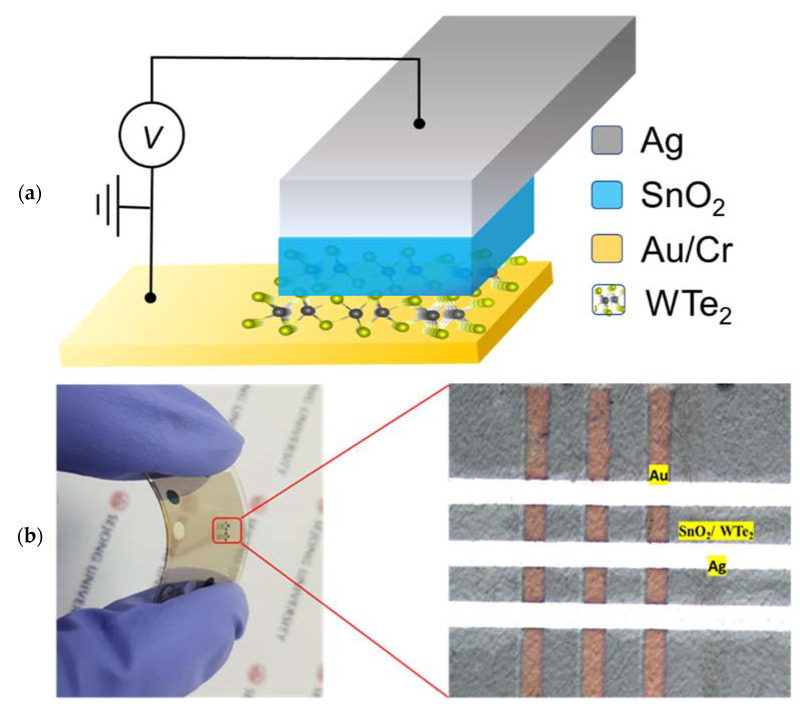
(**a**) Schematic illustration of the resistive-switching devices composed of bilayer SnO_2_/WTe_2_ sandwiched between Ag and Au metal electrodes. Measurement geometry of the electrical connections is also shown for memristive behavior. (**b**) The optical image of the memristive device is also illustrated and marked with yellow boxes.

**Figure 2:**
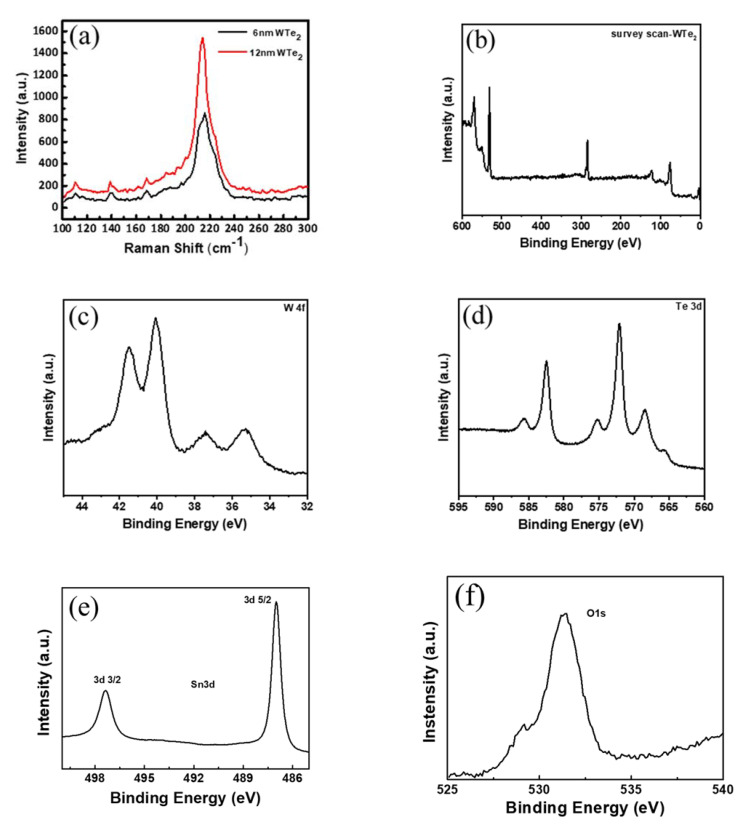
(**a**) Raman Spectroscopy of WTe_2_ on a flexible PET substrate with 6 nm and 12 nm thickness. (**b**) XPS spectra of W and Te elements in WTe_2_ films. (**c**) W 4f level and (**d**) Te 3d level in WTe_2_ films indicating the presence of non-stoichiometric WO_x_ films under the influence of reactive Ag top electrode. (**e**,**f**) The XPS analysis from the surface of the SnO_2_ depicting the Sn3d and O1s peaks.

The fabrication of the device was accomplished by depositing a bottom electrode of Au/Cr (20/5 nm) over a flexible PET substrate via a thermal evaporator in a high-vacuum chamber. A metal mask was used over the PET substrate to pattern the bottom electrode. The thin films of the WTe_2_ and SnO_2_ were directly synthesized at room temperature via RF-magnetron sputtering over the Au/Cr electrode. The working pressure during the flow of Argon gas was set to 2 × 10^−3^ torr, while the chamber pressure was set to 3 × 10^−6^ torr. The EDS analysis and elemental mapping are illustrated in Figure S1a–c and Table S1 for WTe_2_ while theEDS analysis, elemental mapping, and their weight percentage ratio for SnO_2_ are illustrated in Figure S2a–c and Table S2. Finally, the silver (Ag) top electrode, as an electrochemically active electrode (50 nm), was grown over it via a thermal evaporator. The schematic diagram is depicted in Figure 1a, illustrating the device geometry. The optical image of the final device is illustrated in Figure 1b. The quality of the 2D WTe_2_ film was verified under Raman spectroscopy analysis and the X-ray photoelectronic spectroscopy (XPS) spectra. In Raman analysis of the WTe_2_ films on the flexible PET substrate, the four prominent peaks, which appear because of in-plane and out-of-plane modes of vibration [23,24], were observed around 110, 142.1, 168.1, and 222 cm^−1^, as shown in Figure 2a. Furthermore, the surface analysis of the synthesized WTe_2_ films was investigated under the XPS to observe the chemical surface states, as illustrated in Figure 2b. The initial four characteristic peaks [25,26] belong to the tungsten metal W (4f), while the rest of the characteristic peaks belong to the Te (3d), as shown in Figure 2c,d. The XPS spectra for the SnO_2_ are presented in Figure 2e,f. Two characteristic peaks for Sn (3d) and one characteristic peak for O (1s) were observed during the XPS analysis, which confirms the quality of the synthesized SnO_2_. To measure the resistive-switching behavior of the Ag/SnO_2_/WTe_2_/Au devices, the direct current (DC) voltage pulses were applied to the top Ag electrode, and the bottom Au electrode was grounded. The I-V characteristics were studied at room temperature by using the Agilent B1500 semiconductor parametric analyzer in its DC sweep mode.

## 3. Results and Discussions

**Figure 3:**
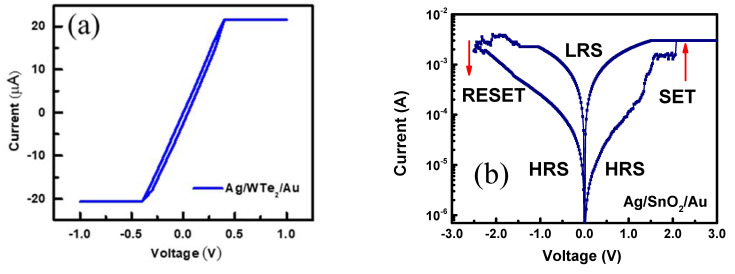

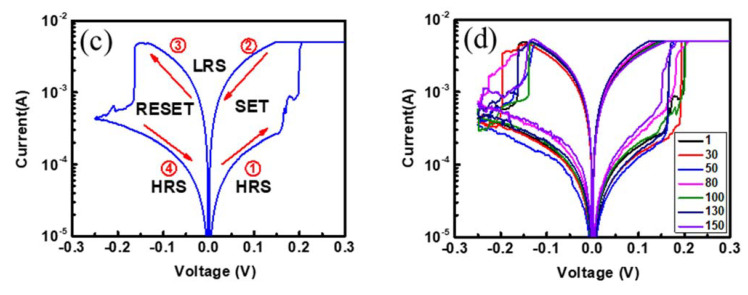
(**a**) The I-V curve under a compliance current limit, showing Ohmic behavior with WTe_2_ film. (**b**) I-V characteristics of Ag/SnO_2_/Au memristive structure with high RESET and SET voltages. (**c**) I-V characteristics of Ag/SnO_2_/WTe_2_/Au memristive structure with RESET and SET states. (**d**) The consecutive 150 cycles of bilayer memory devices over the flexible PET substrate show high stability.

Initially, the role of the WTe_2_ film is examined via a Ag/WTe_2_/Au device. The current-voltage (I-V) curve extracted from the Ag/WTe_2_/Au shows pure Ohmic behavior, but no resistive switching was observed, as shown in Figure 3a. The resistive switching of the Ag/SnO_2_/Au device is also depicted in Figure 3b. To measure the resistive switching of the Ag/SnO_2_/WTe_2_/Au devices, the bottom, inert Au electrode is grounded, and the top, active Ag electrode is set to apply the voltage bias. To trigger the memory device from an HRS to an LRS, a long voltage sweep is initially required, which forms a conducting filament, as shown in Figure S3. Interestingly, the filament formation voltage (SET) for our Ag/SnO_2_/WTe_2_/Au devices was not as high as those observed in previously reported memristor devices [27,28]. The I-V curves represent the bipolar resistive-switching (RS) characteristics for both the devices Ag/SnO_2_/WTe_2_/Au and Ag/SnO_2_/Au. The I-V curves obtained show that SnO_2_ grown over the WTe_2_ surface enhances the device’s performance and stability after multiple bending cycles. However, while the Ag/SnO_2_/Au devices exhibited stable resistive switching before flexible testing, after multiple bending cycles, it deteriorated the device performance. The flexible nature of the 2D WTe_2_ films means they provide an excellent platform to fabricate flexible memory devices with metal oxides. Several cracks were observed over the surface of the SnO_2_ after bending cycles if it grew directly over the PET substrate, but the SnO_2_ grown over the WTe_2_ illustrated a crack-free surface, as shown in the scanning electron microscopy (SEM) images presented in Figure S4a,b.

Initially, the bilayer structure of the SnO_2_/WTe_2_ was in an HRS, so a large voltage sweep was applied with a compliance current (Icc) limit of 5 mA to avoid the permanent breakdown of the memristive devices. During this voltage sweep, the diffusion of the Ag ions takes place, and their stacking causes filament formation, which shorts the circuit. As the filament forms completely, the device suddenly switches to an LRS and reaches Icc, as shown in Figure 3c. This filament-formation biasing voltage is also marked as a SET voltage, after which a large threshold in current is observed abruptly. Furthermore, when the applied voltage is swept from 0 V to the negative, it suddenly triggers the device to the HRS, which is marked as a RESET voltage. The Ag/SnO_2_/WTe_2_/Au device stability was tested over up to 150 consecutive cycles, which showed its reproducibility over the flexible PET substrate. The I-V curves obtained from the 150 consecutive cycles are illustrated in Figure 3d.

**Figure 4:**
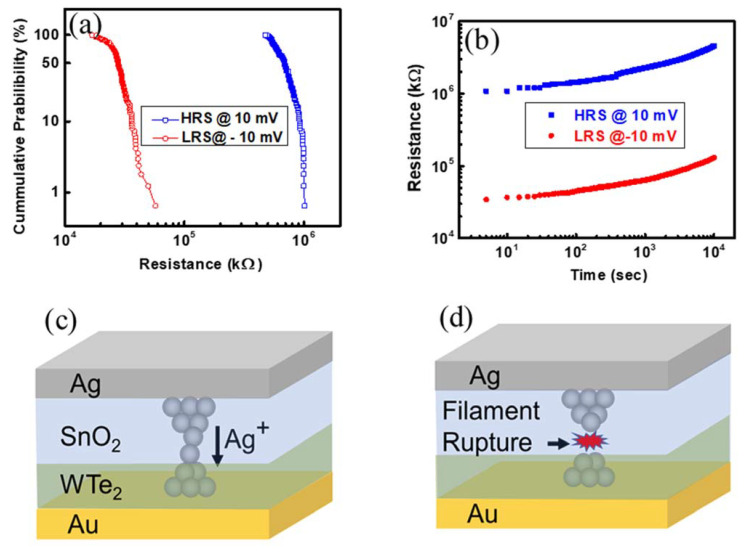
(**a**) The statistical distributions of the resistances of the Ag/SnO_2_/WTe_2_/Au stack memory device under consecutive DC I-V sweep. (**b**) Retention characteristics over 10^4^ s for resistance states. (**c**) The filament formation under the applied voltage bias is due to the diffusion and stacking of the Ag ions. (**d**) The filament rapture state when an opposite polarity is applied to SET the device.

The statistical variation in device resistance as per the number of measurement cycles is plotted in Figure 4a. The Ag/SnO_2_/WTe_2_/Au device was tested for the consecutive 150 cycles, and its LRS and HRS are plotted together. The cumulative probability for 150 cycles was plotted as the function of the device resistances to examine the endurance of the memory devices. The devices showed very stable and repeatable resistive levels of the HRS and LRS for each cycle with a stable rectification ratio. The device retention is also illustrated in Figure 4b, in which the device’s resistive levels are plotted as the function of the time. There was no significant variation in the extracted HRS and LRS values, which confirms the device’s stability over the flexible PET substrate. Furthermore, the physical mechanism behind the RESET and SET state of the device was elaborated in detail. The HRS level was achieved in all fabricated devices over the PET substrate. However, evolution from an HRS to an LRS during the SET process occurs due to the sudden enhancement of current up to Icc when the sweeping voltage reaches the SET voltage (V_SET_). At LRS, the current which runs from the device is higher because of the high conductivity of the Ag metallic filaments. In hybrid devices, this switching mechanism occurs just by swapping the stacking sequence of the solid electrolyte. Moreover, in hybrid devices, RS characteristics perform better results as compared to single-thin-film devices. The reactions possible for the formation/rupture of the Ag electrochemically active electrode filaments in the active layer during the SET/RESET process are shown below.

Ag Ag^+^ + 1e^−^ (Oxidation) Ag^+^ + 1e^−^ Ag (Reduction)

As an outcome of this redox reaction, the metal ions (Ag+) drift through the insulator (switching material) to form the conduction filament. Owing to a positive bias voltage applied to the top electrode during the SET process, the Ag ion isolates from the top electrode, travels through the switching material, and decisively settles down as an Ag atom at the bottom electrode after being reduced. In this approach, Ag filaments form and make the connection between the top and bottom electrodes. Prior studies have explained that ion migration and redox reaction rates are the reason why several conduction filament modes are predicted [29]. A nanocluster of Ag nucleates moves through the electrolyte when an external bias is applied. Moreover, overall ion migration is caused by the movement of the nanocluster. Contrarily, when the top electrode is negatively biased, the filaments formed during the SET process are ruptured. The physical mechanism of filament formation and its rupture is illustrated in Figure 4c,d. The electric field track and the redox reaction that occur at the electrode are liable for successful ion drift [29]. Moreover, if the redox reaction rate is high but the ion migration rate is low, then the filament formation materializes inside the dielectric material and on the cathode side of the filament; a large amount of metal is hoarded in this situation. This is an uncompleted filament after the initial filament formation. In a reverse condition, where the redox reaction is lower and the migration rate is higher, a branched cut filament is produced from the inert to active electrode. The inadequate supply of ions is the fundamental reason why the reduction reaction occurs at the edges of the filament [29]. The Ag ion transport rate is hindered by the SnO_2_ layer, causing a low ion transport rate in the oxidized layer. However, for the formation of the conductive filaments, the nucleation or the growth of the Ag filaments must be balanced for its continuous growth during the SET process. This effect is balanced by inserting the WTe_2_ layers. The Ag ion transmission rate is relatively high in 2D materials. The movement process of the Ag ions in a 2D layer corresponds to the forming process. So, the use of a 2D interfacial layer results in highly stable memristive switching and highly concentrated SET/RESET voltage distribution. After the filament reaches the Au bottom electrode, the device reaches its ON-state. In addition, the weakest link of the Ag conductive filament happens at the 2D material and oxide layer interface, which is the reason an abrupt rupture has been observed during the reverse bias, whereas for the single-layer SnO_2_ memory stack, gradual switching with a high RESET and low ON/OFF ratio has been observed. This low ON/OFF ratio (4 × 10^2^) of the Ag/SnO_2_/Au devices is possibly attributed to the defect states; hence, the use of a 2D-based bilayer stack results in the drastic improvement of the switching parameters. Secondly, Schottky emission also occurs because of thermal activation. When the positive bias is applied to the top Ag electrode, thermally activated electrons receive a sufficient amount of energy to jump from the valence band to the conduction band [30,31,32]. Therefore, it can be concluded that different material devices with diverse geometry and parameters can provide an outcome of conducting filament occurrence conditional to the ion migration rate and redox reaction. At the electrode, the migration and redox reaction rate of an Ag ion fluctuates depending upon the physical and chemical properties of different materials.

## 4. Conclusions

In summary, we synthesized a bilayer structure of tin-oxide/tungsten-ditelluride (SnO_2_/WTe_2_), sandwiched between Ag (top) and Au (bottom) metal electrodes over a flexible PET substrate. The flexible nature of the two-dimensional WTe_2_ was utilized to achieve a better platform for the SnO_2_ to fabricate a highly stable flexible memory device. The flexible memory device is triggered from a high-resistance state (HRS) to a low-resistance state (LRS) by filament formation between the top and bottom electrodes, attributed to the diffusion of the Ag ions. The filament formation, which is responsible for the resistive switching, has been explained with its underlying mechanism of ion diffusion and Schottky emission. The repeatable cycles of resistive switching are reported with the bilayer structure of SnO_2_/WTe_2_. A few layers of a thick film of WTe_2_ provide highly repeatable resistive-switching results with a low switching voltage. The Ag/SnO_2_/WTe_2_/Au devices exhibited excellent retention and bending endurance. This research might lead to the development of highly stable and flexible resistive-switching devices for the next generation of wearable electronics.

## Data Availability

Not applicable.

## Supplementary Materials

The following are available online at https://www.mdpi.com/article/10.3390/ma14247535/s1, Figure S1: (a,b) The elemental mapping of the W and Te elements are presented with a scale bar of 1 um. (c) The corresponding peaks belonging to W and Te are presented in yellow color, Figure S2: (a,b) The elemental mapping of the Sn and O is presented with a scale bar of 1 um. (c) The corresponding peaks belonging to Sn and O are presented in yellow color, Figure S3: The filament formation is illustrated during the 1st sweep which triggered the device from HRS to LRS, Figure S4: (a) The SEM image of the SnO_2_ film showing cracks and (b) bilayer SnO_2_/WTe_2_ cracks free film over the flexible PET substrate after the 100 bending cycles, Figure S5: (a) Optical image of flexible bilayer memristive structure based on flexible PET substrates, and (b) displaying its transmittance measurements, Table S1: The atomic and weight percentage of each element present in WTe_2_ is illustrated, Table S2: The atomic and weight percentage of each element present in SnO_2_.
