# Supplementary material for: Flexible Memory Device Composed of Metal-Oxide and Two-Dimensional Material (SnO2/WTe2) Exhibiting Stable Resistive Switching
Source: Materials (Basel). 2021 Dec 8;14(24):7535. doi: 10.3390/ma14247535 (PMC8708916; doi:10.3390/ma14247535)
Supplement: Supplementary file 1 [file materials-14-07535-s001.zip › materials-1493162-supplementary.pdf]

Supporting Information

Flexible and highly stable bilayer structure of (SnO_2/WTe_2) metal-oxide and two-dimensional material exhibiting excellent resistive switching.

Ghulam Dastgeer[1*], Amir Muhammad Afzal[2], Jamal Aziz[3], Sajjad Hussain[4], Syed Hassan Abbas Jaffery[4], Deok-kee Kim[3], Muhammad Imran[5], Mohammed A. Assiri[5]

[1]Department of Physics & Astronomy and Graphene Research Institute, Sejong University, Seoul 05006, Korea.
[2]Department of Electrical and Biological Physics, Kwangwoon University, Seoul 01897, South Korea.
[3]Department of Electrical Engineering, Sejong University, 209 Neungdong-ro, Gwangjin-gu, Seoul 05006, South Korea
[4]HMC (Hybrid Materials Center), and Department of Nanotechnology & Advanced Materials Engineering and Graphene Research Institute, Sejong University, Seoul, 05006 Korea.
[5]Department of Chemistry, Faculty of Science, King Khalid University, P.O. Box 9004, Abha 61413, Saudi Arabia.

Corresponding Author: Ghulam Dastgeer

Email: gdastgeer@sejong.ac.kr

Energy-dispersive X-ray spectroscopy (EDX) analysis of the WTe_2 and SnO_2.

To characterize the thin films based on bilayer structure which comprises WTe_2 and SnO_2, the energy-dispersive X-ray spectroscopy (EDX) analysis is performed[1]. The elemental mapping of the W and Te elements are presented in **Figure S1a-b**, while the corresponding peaks confirming the presence of W and Te are shown in **Figure S1c**.

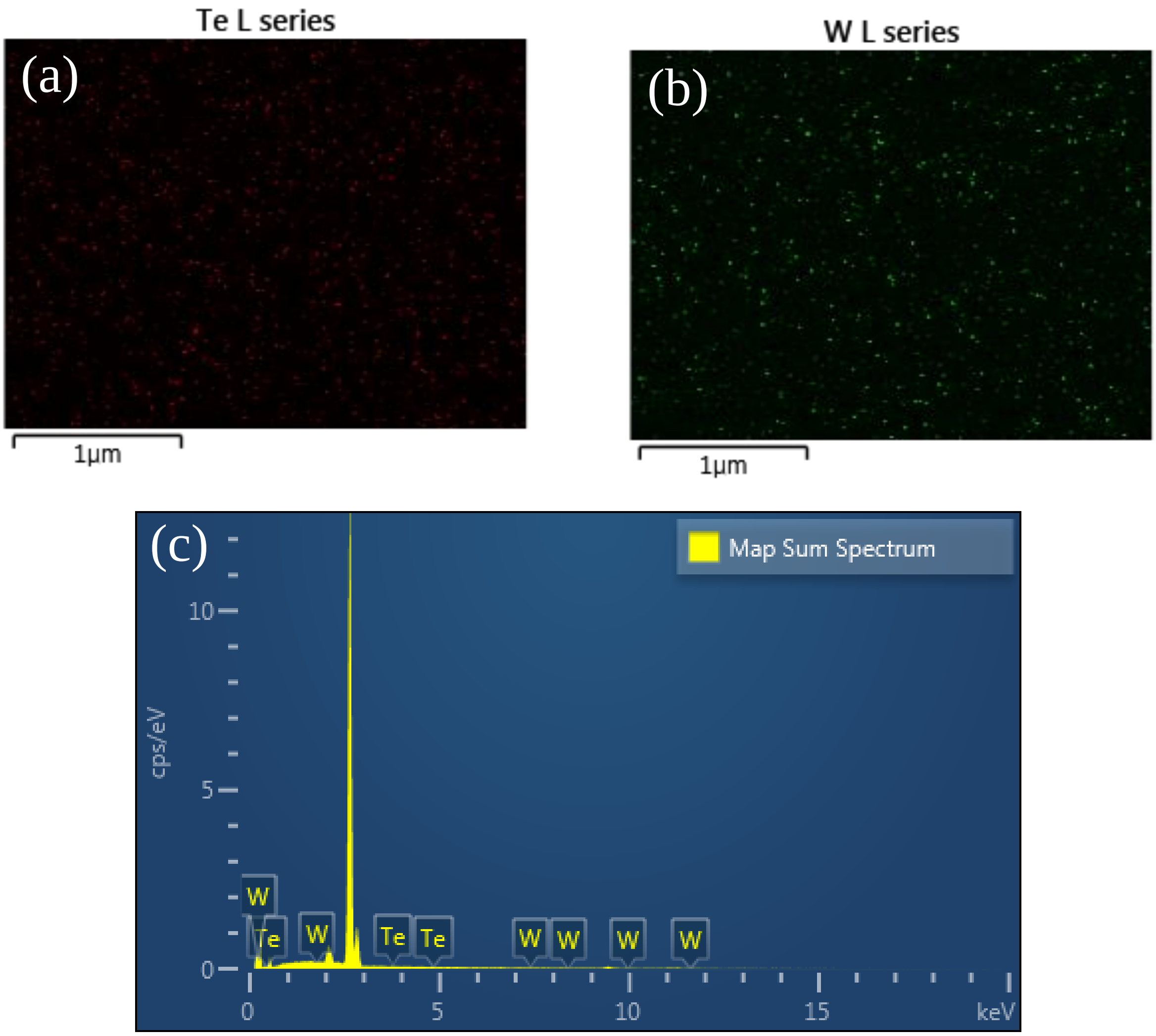

Figure S1. (a,b) The elemental mapping of the W and Te elements are presented with a scale bar of 1 um. **(c)** The corresponding peaks belonging to W and Te are presented in yellow color.

Table S1. The atomic and weight percentage of each element present in WTe_2 is illustrated.

Element	Line Type	Apparent Concentration	k Ratio	Wt.%	Wt.% Sigma	Standard Label	Factory Standard
Te	L series	1.45	0.01120	55.98	6.96	HgTe	Yes
W	L series	1.14	0.01139	44.02	6.96	W	Yes
Total:				100.00			

The EDX analysis of all the elements present in SnO_2 is examined by element mapping and their corresponding peaks are shown in Figure S2a-c. The atomic and weight percentage of each element is presented in Table S2.

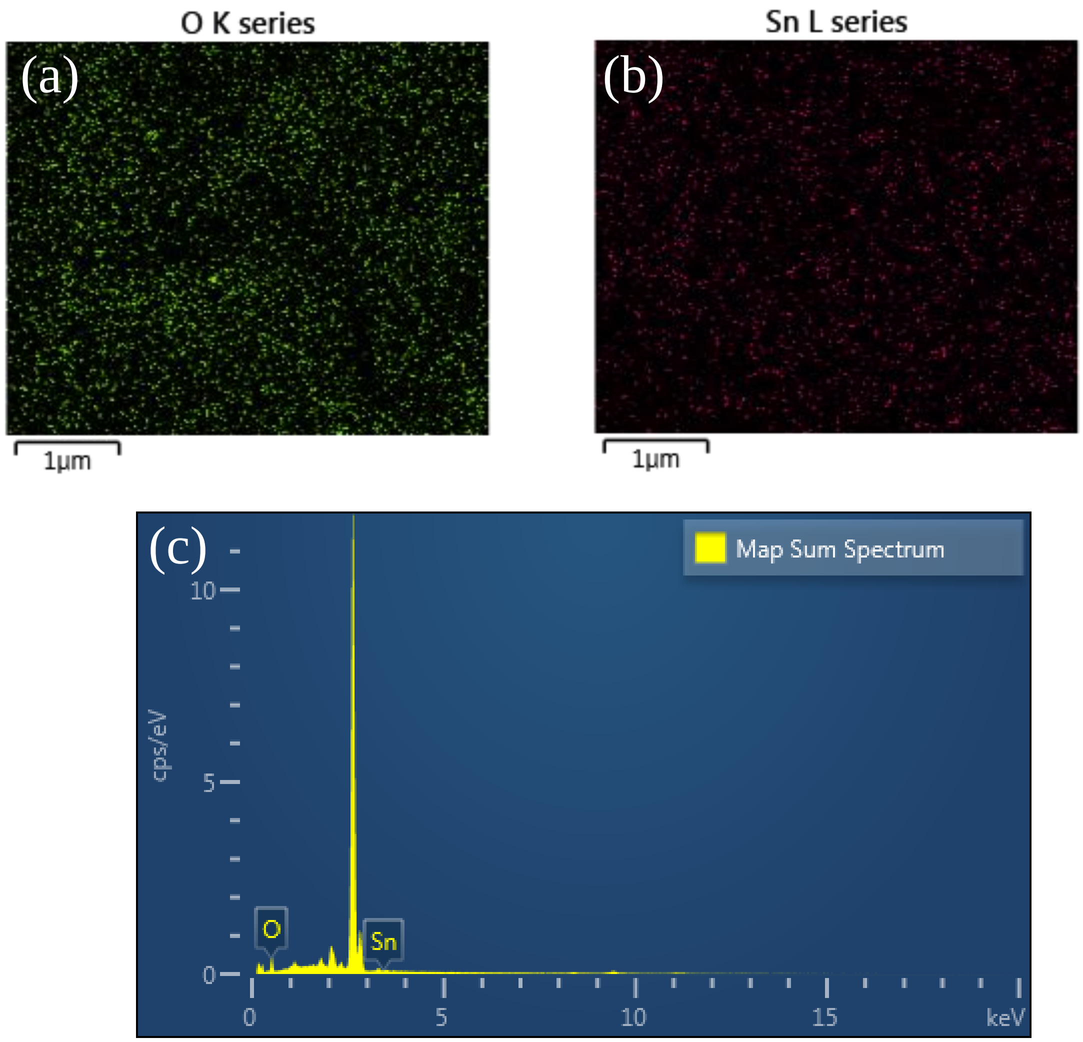

Figure S2. (a,b) The elemental mapping of the Sn and O is presented with a scale bar of 1 um. **(c)** The corresponding peaks belonging to Sn and O are presented in yellow color.

Table S2. The atomic and weight percentage of each element present in SnO_2.

Element	Line Type	Apparent Concentration	k Ratio	Wt.%	Wt.% Sigma	Standard Label	Factory Standard
O	K series	3.77	0.01270	100.00	0.00	SiO2	Yes
Sn	L series	0.00	0.00000	0.00	2.62	Sn	Yes
Total:				100.00			

Electroforming Process:

The filament formation and the electroforming cycle during the 1st voltage sweep are illustrated for the Ag/SnO_2/WTe_2/Au device in Figure S3. Generally, the electroforming process is required to initiate the resistive switching process[2]. As the high positive biasing voltage is applied to the top Ag top electrode, it starts to diffuse the Ag++ ions through the switching material and form conducting filaments. As the filament formed, the device suddenly triggered from HRS to LRS.

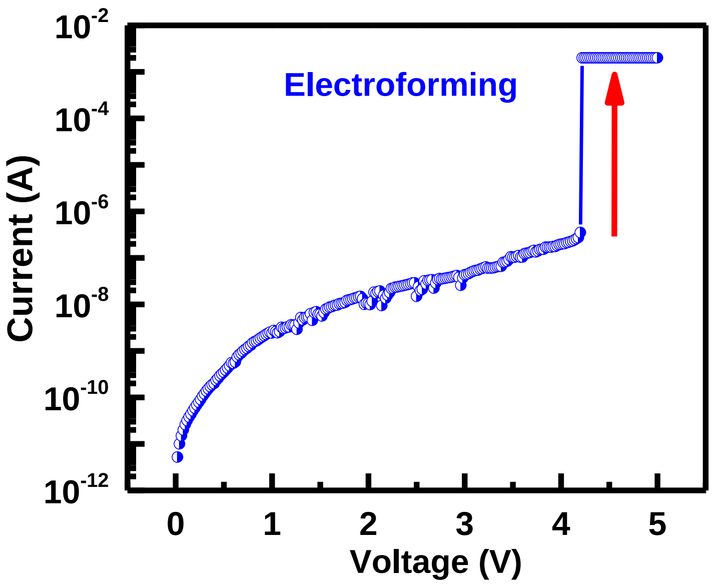

Figure S3. The filament formation is illustrated during the 1st sweep which triggered the device from HRS to LRS.

Scanning Electron Microscopic Analysis:

The scanning electron microscopy (SEM) analysis is performed for both SnO_2 and SnO_2/WTe_2 films over the flexible PET substrate after 100 bending cycles as shown in Figure S3a and Figure S3b, respectively. The SEM images illustrate the fact that there are negligible cracks and fractures in the bilayer SnO_2/WTe_2 film as compared to the directly grown SnO_2 film over the PET substrate as shown in **Figure S4a-b**. The formation of crack-free surfaces for the bilayer structure than the single SnO_2 stack results in the stable resistive switching devices over consecutive I-V sweeps, which is presented in the main manuscript.

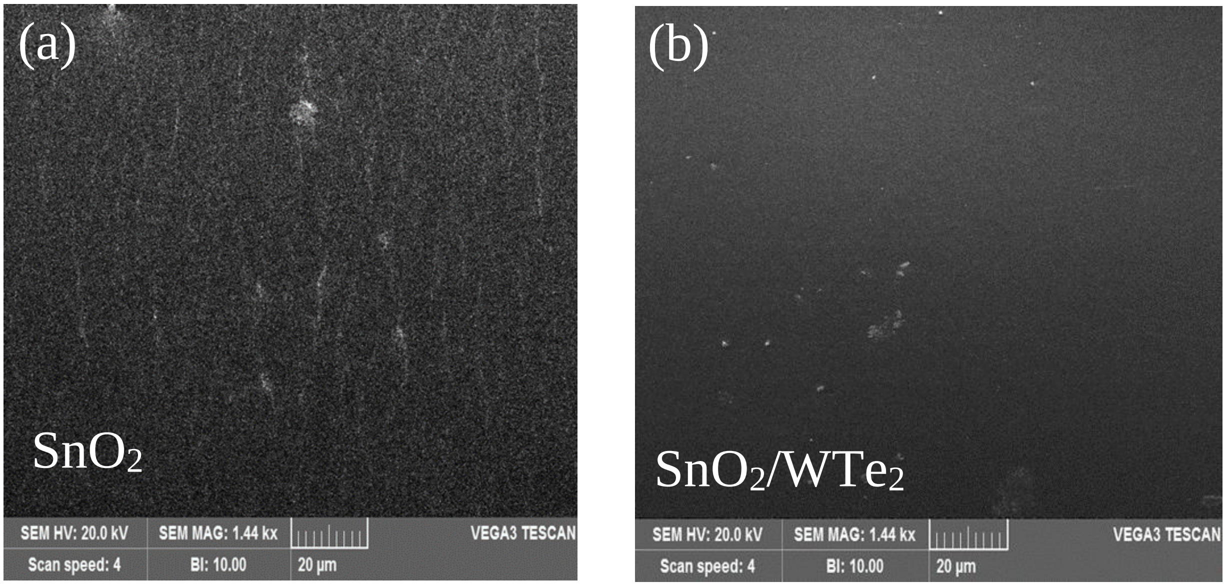

Figure S4. **(a)** The SEM image of the SnO_2 film showing cracks and **(b)** bilayer SnO_2/WTe_2 cracks free film over the flexible PET substrate after the 100 bending cycles.

Transmittance spectroscopy of the bilayer SnO_2/WTe_2 based switching devices utilized in this study.

The bilayer structures fabricated in this study retain average optical transmittance over 30 % in the visible region (400-800 nm) fabricated on the flexible PET substrates. Conventionally, the absorbance spectra are divided into two bands. One emission corresponds to the band edge emission (charges relax from conduction band (C.B) to valence band (V.B)). The second band corresponds to defect edge emission (charges relax from defect states to valence band (V.B)). The transmittance spectra shown in **Figure S5b** presents a peak at 350 nm. As a wide bandgap semiconductor, SnO_2 has a bandgap of 3.6 eV at room temperature which is equivalent to 344 nm. No defect-related peak was observed in such devices. Whereas 2D WTe_2 is expected to have the narrowest bandgap and exhibits the optical absorbance in the Infrared region. The flexible and transparent memristive devices fabricated in this study presents advances in the multi functionalities of RRAM.

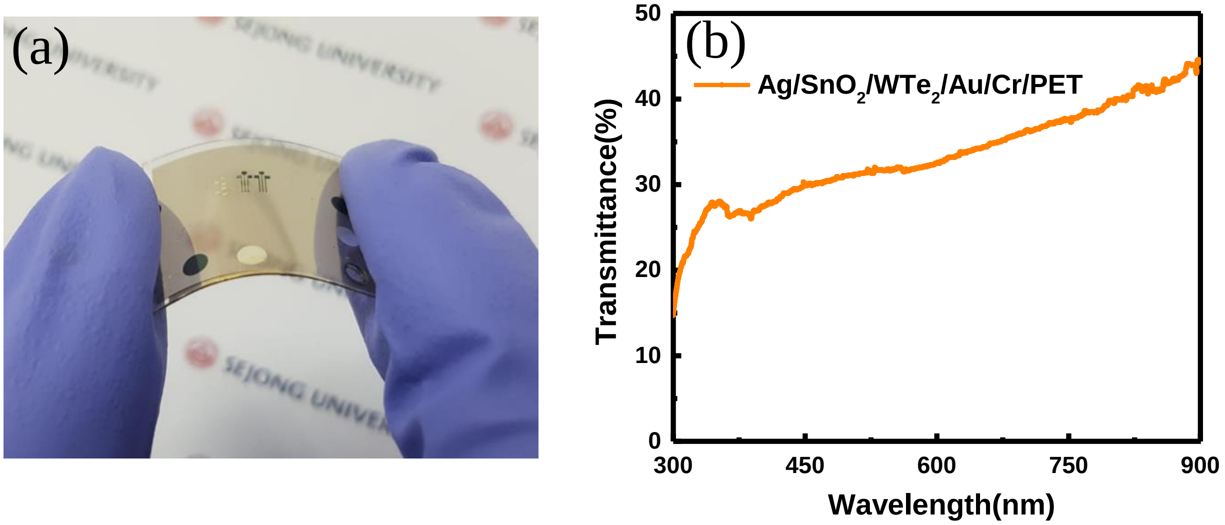

Figure S5. (a) Optical image of flexible bilayer memristive structure based on flexible PET substrates, and **(b)** displaying its transmittance measurements.

References:

1. Buchkov, K.; Todorov, R.; Terziyska, P.; Gospodinov, M.; Strijkova, V.; Dimitrov, D.; Marinova, V., Anisotropic Optical Response of WTe2 Single Crystals Studied by Ellipsometric Analysis. *Nanomaterials* **2021,** *11* (9).
2. Shen, Z.; Zhao, C.; Qi, Y.; Xu, W.; Liu, Y.; Mitrovic, I. Z.; Yang, L.; Zhao, C., Advances of RRAM Devices: Resistive Switching Mechanisms, Materials and Bionic Synaptic Application. *Nanomaterials* **2020,** *10* (8).
